# Protective Role of p70S6K in Intestinal Ischemia/Reperfusion Injury in Mice

**DOI:** 10.1371/journal.pone.0041584

**Published:** 2012-07-27

**Authors:** Kechen Ban, Rosemary A. Kozar

**Affiliations:** Department of Surgery, University of Texas Health Science Center at Houston, Houston, Texas, United States of America; Universidade de Sao Paulo, Brazil

## Abstract

The mTOR signaling pathway plays a crucial role in the regulation of cell growth, proliferation, survival and in directing immune responses. As the intestinal epithelium displays rapid cell growth and differentiation and is an important immune regulatory organ, we hypothesized that mTOR may play an important role in the protection against intestinal ischemia reperfusion (I/R)-induced injury. To better understand the molecular mechanisms by which the mTOR pathway is altered by intestinal I/R, p70S6K, the major effector of the mTOR pathway, was investigated along with the effects of rapamycin, a specific inhibitor of mTOR and an immunosuppressant agent used clinically in transplant patients. In vitro experiments using an intestinal epithelial cell line and hypoxia/reoxygenation demonstrated that overexpression of p70S6K promoted cell growth and migration, and decreased cell apoptosis. Inhibition of p70S6K by rapamycin reversed these protective effects. In a mouse model of gut I/R, an increase of p70S6K activity was found by 5 min and remained elevated after 6 h of reperfusion. Inhibition of p70S6K by rapamycin worsened gut injury, promoted inflammation, and enhanced intestinal permeability. Importantly, rapamycin treated animals had a significantly increased mortality. These novel results demonstrate a key role of p70S6K in protection against I/R injury in the intestine and suggest a potential danger in using mTOR inhibitors in patients at risk for gut hypoperfusion.

## Introduction

Intestinal ischemia/reperfusion (I/R) contributes to the pathogenesis of multiple organ dysfunction syndrome [1]–[3], the leading cause of late deaths in critically ill patients [4]. Intestinal I/R also accompanies a number of clinical and pathophysiological conditions such as trauma, hemorrhage, small bowel transplantation, and cardiopulmonary bypass. In the abdomen, the small bowel is the most sensitive to I/R- induced damage [5]. Acute mesenteric ischemia is reported to have a poor prognosis [6] and the reported incidence of intestinal ischemia is increasing [7]. The tissue is progressively injured during ischemia, but paradoxically, reperfusion further damages the tissue. Reperfusion injury ensues when the restoration of blood flow triggers an intense inflammatory response in organs not involved in the initial ischemic insult, thus resulting in multiple organ dysfunction [8].

Mammalian target of rapamycin (mTOR) complexes (mTORCs) include mTORC1 and mTORC2 [9]. They are functionally distinct. mTORC1 is highly sensitive to rapamycin whereas mTORC2 is insensitive to rapamycin. mTORC1 is consist of mTOR, regulatory-associated protein of mTOR, mLST8, and proline-rich Akt substrate 40. The mTOR pathway phosphorylates ribosomal protein S6 kinase (p70S6K) to activate protein biosynthesis and phosphorylates eukaryotic initiation factor 4E-binding protein 1, subsequently, activated eukaryotic initiation factor 4E to promote protein translation [10]. mTOR therefore is particular important in the regulation of cellular growth, proliferation, cell cycle control, differentiation, motility, survival and in directing immune responses [11]–[14]. It has been implicated in neutrophil, monocyte, dendritic cell, B cell and T cell function [14]–[16]. Inhibition of this pathway enhanced the productions of pro-inflammatory cytokines IL-12 and IL-1β, reduced the productions of the anti-inflammatory cytokine IL-10, and enhanced MHC antigen presentation in dendritic cells and monocytes/macrophages [17]. The mTOR pathway is activated by growth factors and nutrients [18].

As the intestinal epithelium displays rapid cell growth and differentiation and is an important immune regulatory organ [19], we hypothesized that mTOR may play an important role in the protection against intestinal I/R-induced injury. To better understand the molecular mechanisms by which the mTOR pathway is altered by intestinal I/R, p70S6K, the major effector of the mTOR pathway, was investigated along with the effects of rapamycin, a specific inhibitor of mTOR and an immunosuppressant agent used clinically in transplant patients.

## Results

### p70S6K Promoted Intestinal Cell Growth, Decreased Cell Apoptosis and Enhanced Cell Migration in vitro

**Figure 1:**
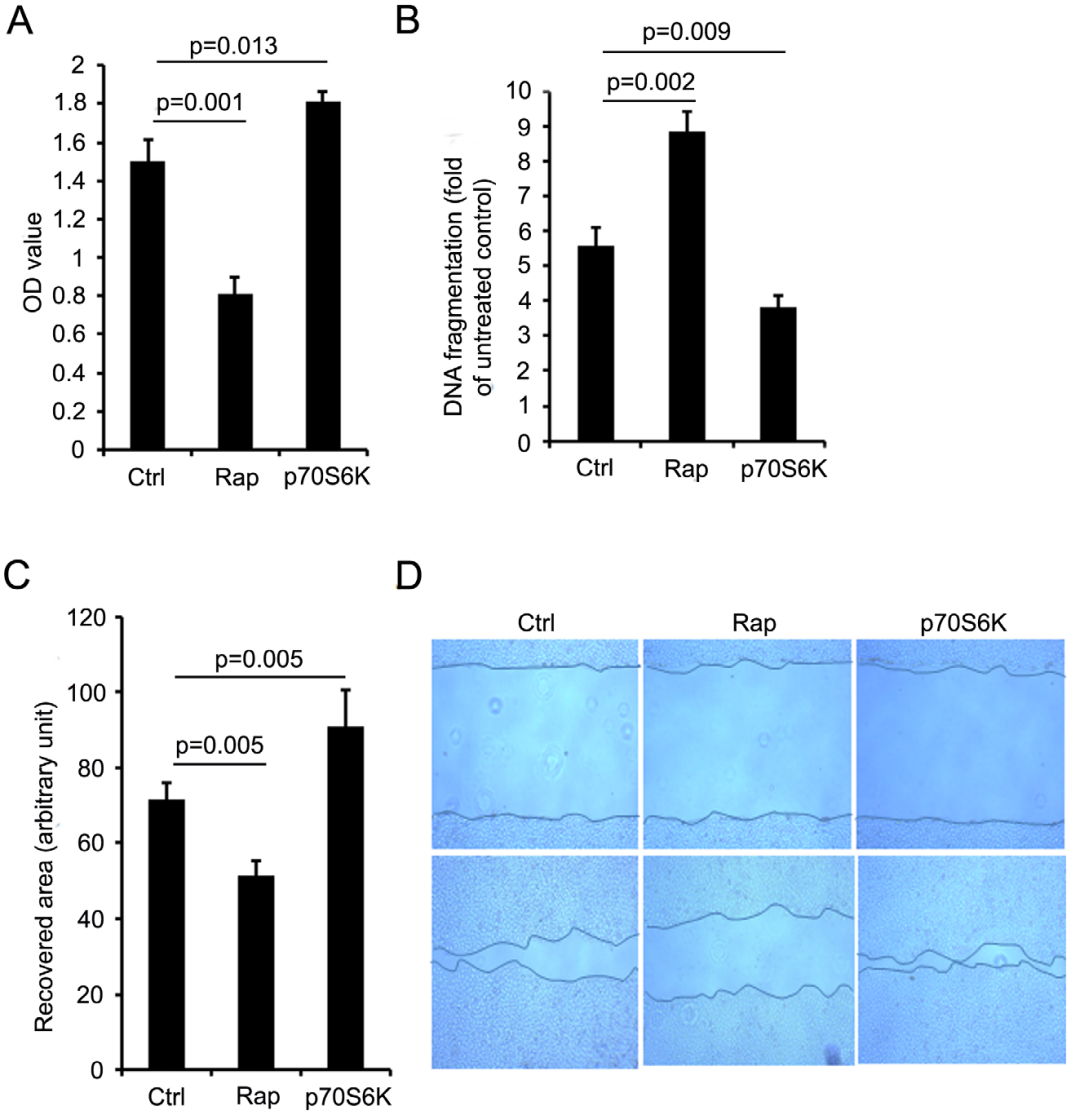
The effect of p70S6K on cell growth, apoptosis and migration. p70S6K expressing plasmid (p70S6K group) and empty plasmid (Ctrl and Rap groups) transfected cells were cultured with FBS-free medium overnight under hypoxic conditions then either treated with vehicle (Ctrl and p70S6K groups) or rapamycin (100 nM) (Rap group) under normoxic or hypoxic conditions. A. Cell growth was measured using MTTassay. B. DNA fragmentation was then measured using a Cell Death Detection ELISA kit. C. Cell migration after wounding. Recovered surface area 24 h after wounding was calculated and quantitative results shown. D. Typical microscopic images of the wound area. The patterns of initial wounding (upper panel) and after 24 h (lower panel) are shown. Black lines indicate the margin of cell migration. All experiments were carried out triplicate. Results are mean ± SEM, p<0.05 statistically significant.

In response to growth factors and nutrients, the mTOR pathway regulates cell proliferation and growth [18]. After hypoxia/reoxygenation, cell growth was significantly decreased by rapamycin (0.81±0.09) compared to vehicle treated controls (1.50±0.11) (p<0.01) but increased in p70S6 overexpressing cells (1.81±0.06) (p<0.05) (Figure 1A). These results indicate that p70S6K increases cell growth.

Although it is well-established that p70S6K plays a crucial role in cell apoptosis, both apoptotic enhancement and protection have been reported [20], [21]. DNA fragmentation, a key feature of apoptosis, was markedly increased after hypoxia/reoxygenation in rapamycin treated cells (8.86±0.59) compared to controls (5.58±0.54) (p<0.01) but decreased in p70S6K overexpressing cells (3.85±0.32) (p<0.01) (Figure 1B), suggesting that p70S6K protects against apoptosis in intestinal epithelial cells cells.

Rapid restitution of the damaged intestinal mucosa depends on intestinal epithelial cell migration to cover the injured areas and p70S6K has been implicated in intestinal epithelial cell migration [22], [23]. As shown in Figure 1C, after hypoxia/reoxygenation cell migration was inhibited in rapamycin treated cells (51.37±3.95) compared to controls (71.52±4.61) (p<0.01) but was enhanced in p70S6K over expressing cells (90.81±9.63) (p<0.05). Representative microscopic images of cell migration are shown in Figure 1D. These data demonstrate that inhibition of p70S6K activity by rapamycin suppressed cell migration, whereas, upregulation of p70S6K activity by plasmid transfection enhanced cell migration in this in vitro wounding model.

### p70S6K was Stimulated in vitro and in vivo

**Figure 2:**
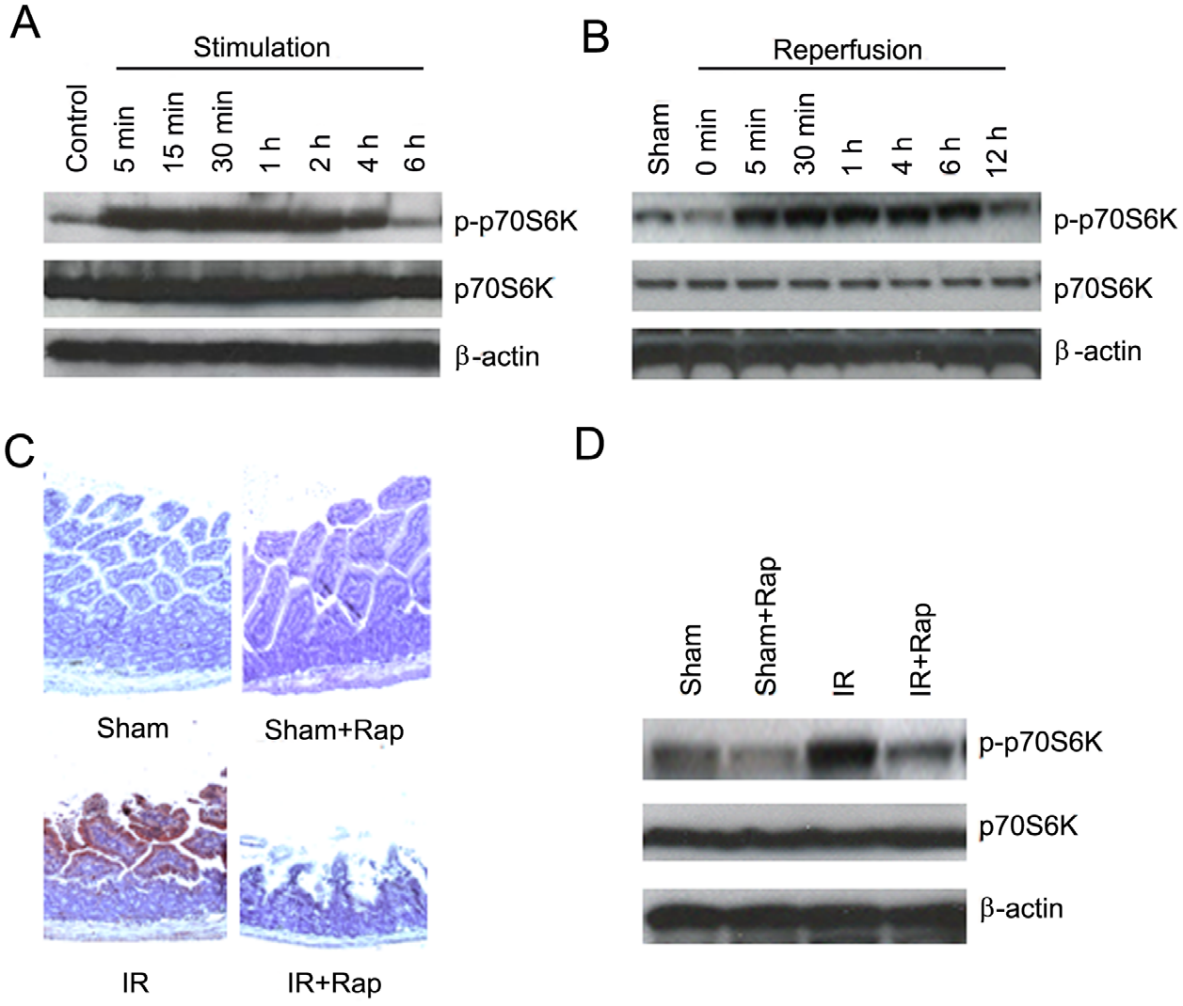
Stimulation of p70S6K in vitro and in vivo and inhibition of p70S6K by rapamycin in vivo. A. Stimulation of p70S6K. Cells were cultured with FBS-free medium overnight under hypoxic conditions and then treated with 10% FBS for indicated time-points under normoxic conditions. Cells were harvested and expression of phosphorylated p70S6K (p-p70S6K) measured. The experiments were carried out triplicate. B. Stimulation of p70S6K by I/R in vivo. The superior mesenteric artery was occluded for 60 min then the intestine was harvested after indicated periods of reperfusion and Western blot analysis performed. C. Confirmation of increased p70S6K activity in intestinal epithelium by immunohistochemistry. Rapamycin or vehicle treated animals underwent intestinal I/R with 6 h of reperfusion. The intestinal tissue was harvested for immunohistochemical staining for p-p70S6K. D. Confirmation of the inhibition of p70S6K by rapamycin using Western blot. Animals were treated as described in Figure 2C. Intestinal tissues were harvested and Western blot analysis performed. For animal experiments, n = 5 mice per group.

After hypoxia/reoxygenation, FBS induced phosphorylation of p70S6K as early as 5 min after stimulation and persisted for 4 h in IEC-6 cells (Figure 2A). Figure 2B demonstrates that in mice, similar to in vitro results, phosphorylation of p70S6K was induced within 5 min of reperfusion, reached the maximal level at 30 min and maintained high level for at least 6 h in the intestine. Immunohistochemical staining demonstrates that the increase of phosphrylation of p70S6K was located in the intestinal epithelium and the increased phosphrylation of p70S6K was significantly inhibited by rapamycin (Figure 2C). Western blot analysis confirmed the increased phosphorylation of p70S6K by I/R was abolished by rapamycin treatment (Figure 2D).

### Inhibition of p70S6K by Rapamycin Worsened Intestinal Damage and Increased Epithelial Cell Apoptosis

**Figure 3:**
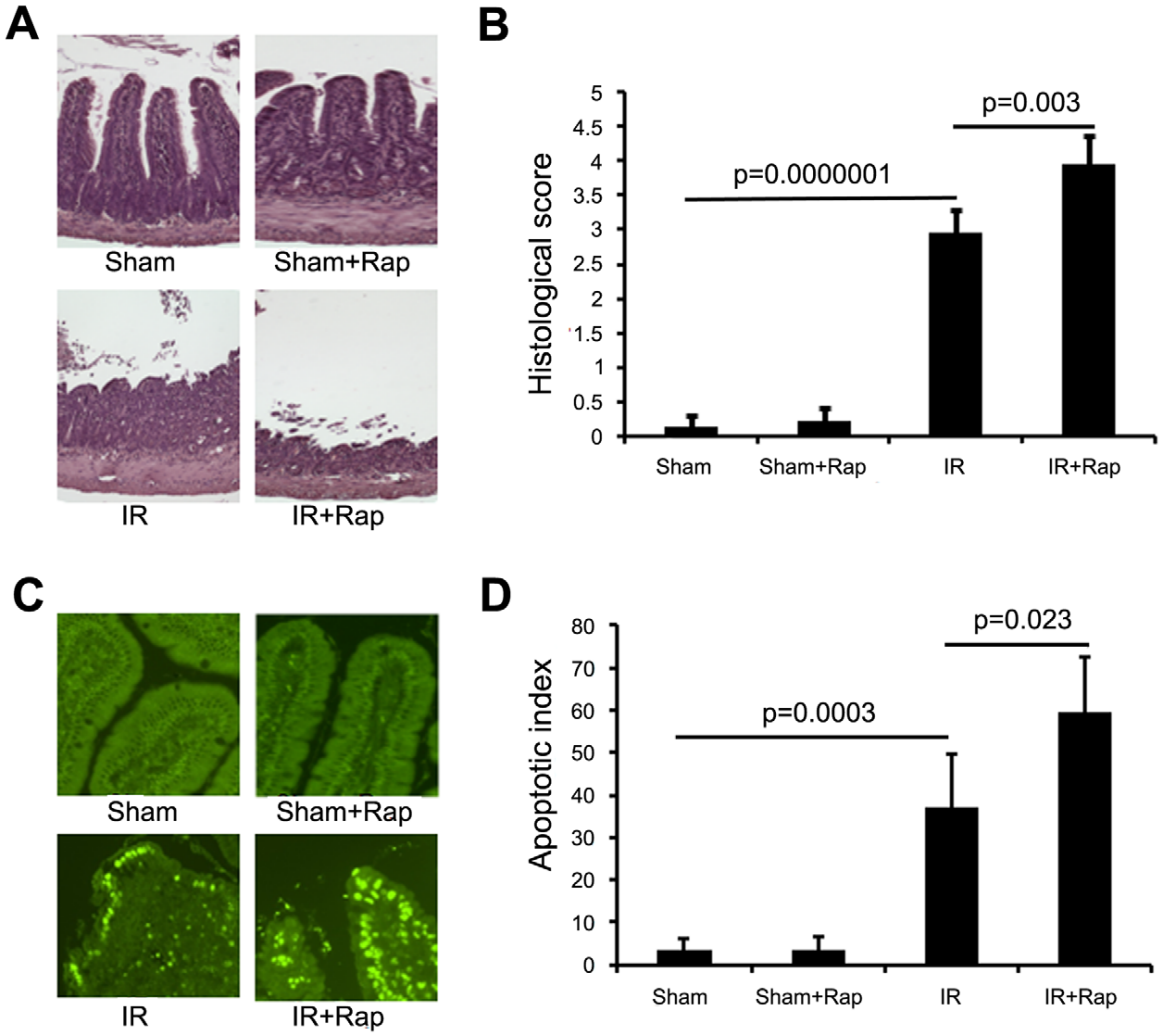
The effect of rapamycin on intestinal damage. Rapamycin or vehicle was administered then intestinal I/R performed with 6 h of reperfusion. A. Representative histological images, magnification x 200. B. Histologic mucosal damage was calculated using the Chiu scoring system [61]. C. Representative images of TUNEL staining in Intestinal tissues. D. Quantitative results of TUNEL staining. Results are mean ± SEM, p<0.05 statistically significant, n = 5 mice per group.

Histopathologic analyses confirmed that I/R resulted in significant mucosal injury compared to shams (2.96±0.34 vs. 0.14±0.17), which was worsened in rapamycin treated mice (3.96±0.42) (Figures 3A and 3B). Comparing to Shams (3.20±2.86), terminal deoxynucleotidyl transferase dUTP nick end labeling (TUNEL) staining also showed apoptotic epithelial cells were markedly increased by I/R (37.0±12.53) and further increased by rapamycin (59.6±12.90) (Figures 3C and 3D). Treatment of rapamycin alone did not significantly affect intestinal damage and epithelial cell apoptosis.

### Inhibition of p70S6K by Rapamycin Promoted Intestinal Inflammation

**Figure 4:**
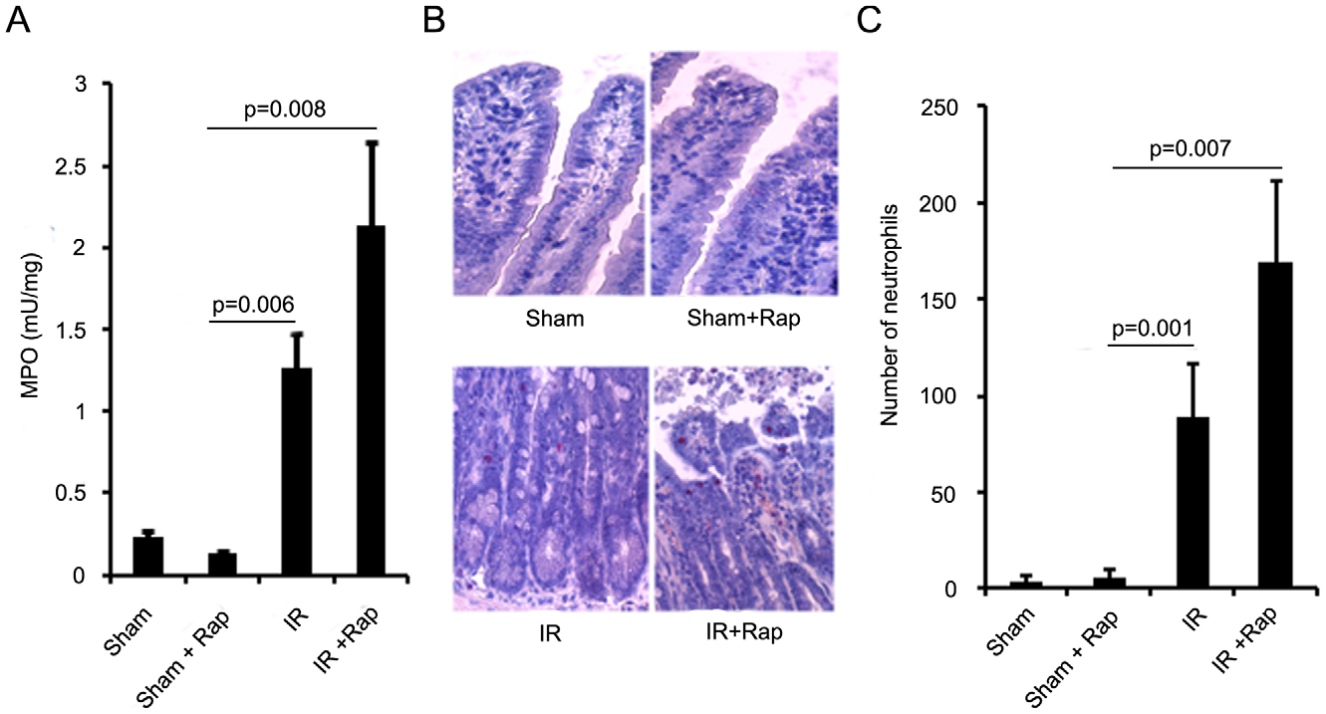
The effect of rapamycin on neutrophil infiltration. Rapamycin or vehicle was administered then intestinal I/R performed with 6 h of reperfusion. A. Myeloperoxidase (MPO) activity in intestinal tissues. B. Representative images of neutrophil staining in intestinal tissues. C. Quantification of positively stained neutrophils. Results are mean ± SEM, p<0.05 statistically significant, n = 5 mice per group.

MPO activity, an indicator of neutrophil accumulation, was evaluated. Compared to shams, a 5.4-fold increase was found in tissue MPO activity in I/R group (Figure 4A). This I/R-induced recruitment of neutrophils was significantly enhanced in rapamycin treated animals (9.2-fold compared to shams), indicating that inhibition of p70S6K by rapamycin enhanced intestinal inflammation. Cytochemical staining also showed I/R resulted in more neutrophil infiltration in intestinal mucosa compared to Shams and additional treatment with rapamycin further enhanced neutrophil infiltration into intestinal mucosa (Figures 4B and 4C). Treatment of rapamycin alone did not significantly affect neutrophil infiltration into intestinal tissues.

### Inhibition of p70S6K by Rapamycin Increased Systemic and Local Pro-inflammatory Cytokines

**Figure 5:**
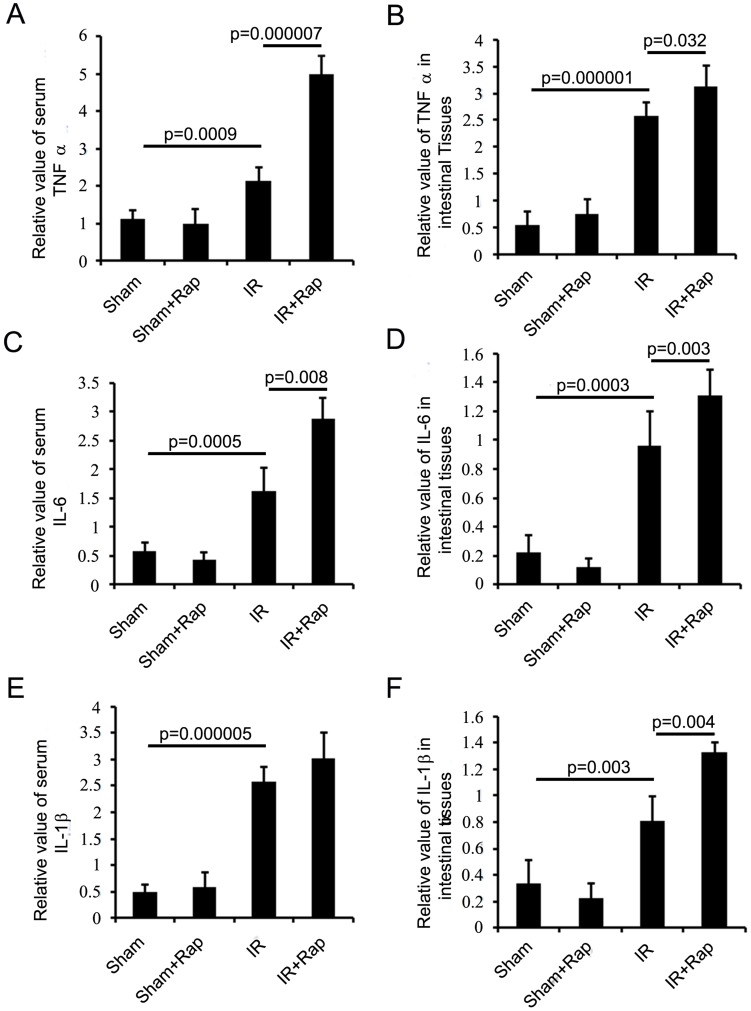
The effect of rapamycin on cytokine levels in serum and intestinal tissues. Rapamycin or vehicle was administered then intestinal I/R performed. Blood and intestinal tissues were collected after 6 h of reperfusion for evaluation of TNF-α, IL-6 and IL-1β levels. A. Relative levels of TNF-α in serum. B. Relative levels of TNF-α in intestinal tissues. C. Relative levels of IL-6 in serum. D. Relative levels of IL-6 in intestinal tissues. E. Relative levels of IL-1β in serum. F. Relative levels of IL-1β in intestinal tissues. Results are mean ± SEM, p<0.05 statistically significant, n = 5 mice per group.

Similar to the findings of Rocourt et al. [24], increased levels of pro-inflammatory cytokines, TNF-α (Figures 5A and 5B) and IL-6 (Figures 5C and 5D), were found after intestinal I/R in both serum and intestinal tissues. Additionally, treatment with rapamycin further increased TNF-α and IL-6. I/R also resulted in an increase of IL-1β levels in both serum and intestinal tissues, but additional treatment of rapamycin further promoted the production of IL-1β only in intestinal tissues but not in serum (Figures 5E and 5F). This further confirmed the pro-inflammatory properties of rapamycin to the hypoperfused gut.

### Inhibition of p70S6K by Rapamycin Enhanced Intestinal Permeability

**Figure 6:**
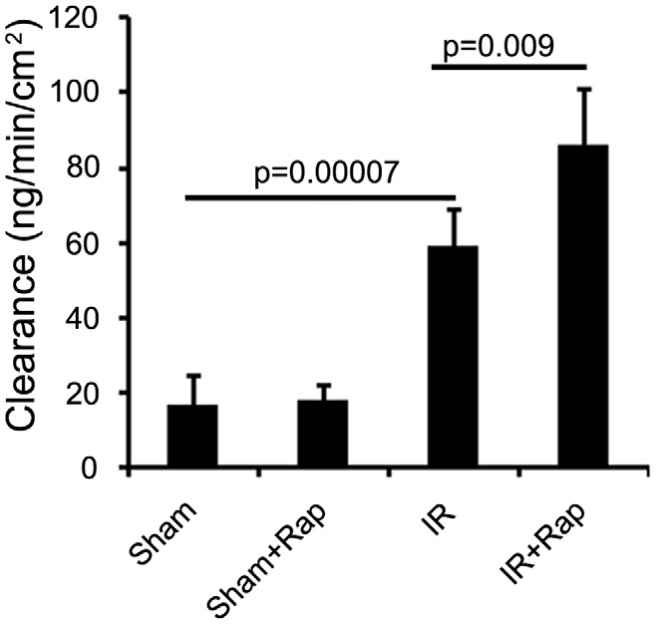
The effect of rapamycin on intestinal permeability. Rapamycin or vehicle was administered then intestinal I/R performed. Intestinal tissues were harvested for permeability using the ex-vivo isolated everted ileum sac method [64]. Intestinal permeability was expressed as the mucosal-to-serosal clearance of FD4. Results are mean ± SEM, p<0.05 statistically significant, n = 5 mice per group.

Gut I/R leads to gut barrier disruption, of which permeability is one manifestation [25]. Loss of normal intestinal barrier function is a key element in the paradigm of gut-origin systemic inflammatory response syndrome and organ dysfunction. Our results show that intestinal permeability is enhanced after I/R compared to shams (59.4±9.65 vs. 16.8±8.11), and further increased by rapamycin (86.2±14.77) (Figure 6).

### Inhibition of p70S6K by Rapamycin Decreased Survival

**Figure 7:**
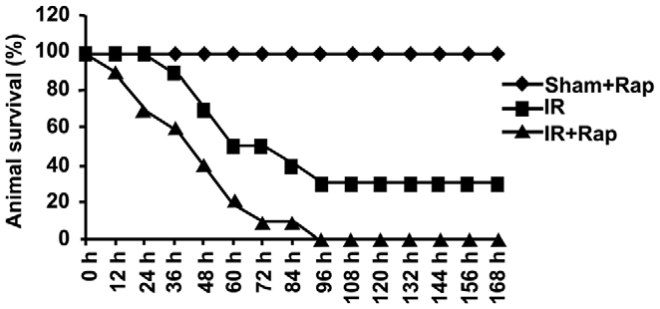
The effect of rapamycin on animal survival. Rapamycin or vehicle was administered then intestinal I/R performed. Animals were observed for 7 days. Survival was analyzed using the Mantel–Haenzel log rank test. P values <0.05 were considered significant. n = 10 mice per group. There was a significant decrease in survival in rapamycin treated animals (p<0.01).

There was a significant decrease in survival in animals that received rapamycin. By four days following intestinal I/R, no mice were alive in the rapamycin-treated group. In comparison, 30% of animals survived in the vehicle-treated group (p<0.01) (Figure 7). This marked difference in mortality clearly demonstrates the detrimental effect of inhibition of p70S6K by rapamycin to the hypoperfused gut.

## Discussion

Our in vivo and in vitro results clearly demonstrate that the mTOR pathway, via p70S6K, provides protection to the bowel after I/R, a novel observation. Importantly, rapamycin, a specific inhibitor of mTOR used clinically in transplant patients, further increased intestinal damage when administered to the hypoperfused gut. In vitro, p70S6K promoted intestinal cell growth, enhanced cell migration, and reduced apoptosis. These protective effects were abolished by rapamycin. In a mouse model of gut I/R, inhibition of p70S6K by rapamycin worsened gut injury, promoted inflammation, and enhanced intestinal permeability. Importantly, rapamycin treated animals had a significantly increased mortality.

Although it has been well-established that the mTOR pathway plays a crucial role in cell function, its effects on different types of cells can vary. For instance, treatment of rapamycin induced cell apoptosis in a human gastric cancer cell line [26] and a non-small cell lung cancer cell line [27], but caused cell resistance to apoptosis in rat beta cells [28], rat renal tubular epithelial cells [29], and human proximal tubular epithelial cells [30]. p70S6K-deficiency also protected against apoptosis in hepatocytes [31]. In the present study, we found that overexpression of p70S6K accelerated cell proliferation and migration but reduced cell apoptosis, while inhibition of p70S6K by rapamycin showed increased apoptosis, suggesting a protective role of p70S6k in intestinal epithelial cells. In vivo, the effect of rapamycin in I/R injury is also controversial. The beneficial effect of rapamycin in I/R was previously reported in kidney [32]–[34], heart [35], [36], and liver [37], while in other studies the detrimental effect of rapamycin was also reported in kidney [38], [39] and brain [40]. In the present study, inhibition of p70S6K by rapamycin worsened the intestinal damage after I/R. Clearly, p70S6K has distinct functions in different cell types and under different circumstances.

Interestingly, our results showed that despite the well-documented immunosuppressive profile of rapamycin, it enhanced intestinal inflammation during I/R under our experimental conditions. The pro-inflammatory effects of rapamycin are further supported by the increased levels of the pro-inflammatory cytokines, TNF-α and IL-6. The observations that glomerulonephritis, interstitial pneumonitis and lymphocytic alveolitis were found in patients treated with rapamycin also support its pro-inflammatory side effects [17]. The precise mechanisms by which this immunosuppressive agent could elicit pro-inflammation in the hypoperfused gut are not understood. A potential mechanism may be through decreased peroxisome proliferator-activated receptor γ (PPARγ) activity, an anti-inflammatory transcription factor important in intestinal inflammation [41]. mTOR was required for PPARγ activation in the vascular endothelium [42], and rapamycin counteracted PPARγ activation in lipopolysaccharide-stimulated RAW264.7 macrophages [43] as well as inhibited PPARγ expression 3T3-L1 preadipocytes [44]. Previous studies from our and other groups have demonstrated that PPARγ provides protection to the hypoperfused bowel [45]–[48]. A second potential mechanism that could support pro-inflammation by mTOR inhibition is via enhanced transcription nuclear factor-κB (NF-κB) activity [49]. We have found that NF-κB is important in the injurious effects of intestinal I/R [50] and rapamycin has been found to promote production and activity of NF-κB [43], [49].

Fujishiro *et al*. reported that rapamycin lessened leukocyte infiltration and diminished pro-inflammatory mediators in graft intestinal muscularis in a rodent model of small bowel transplantation [51], but inflammation in the mucosa and blood was not observed. Additionally, Puglisi et al. previously reported that rapamycin had beneficial effects on intestinal ischemic injury by demonstrating decreased expression levels of xanthine oxidase and increased expression levels of maltase in intestinal tissues along with decreased systemic TNF-α levels [52]. However, it is possible that these benefits resulted from the inhibition of immune cell proliferation by rapamycin [53], as mice were pretreated with rapamycin for five days prior to ischemia. Rapamycin and its analogues are used after solid organ transplantation, and being tested as therapy to treat solid tumors, coronary restenosis, and rheumatoid arthritis [54]. The gastrointestinal effects of immunosuppressive drugs are also being increasingly appreciated. We have demonstrated that rapamycin has potent deleterious effects on the post ischemic gut, suggesting that its use in patients with gut hypoperfusion should be further examined. These results also highlight the potential danger to the use of mTOR inhibitors as immunosuppressive agents in patients at high risk in the very early phase of intestinal transplantation.

In summary, we were the first to show the critical role of p70S6K in protection against intestinal injury induced by I/R. In vitro, p70S6K promoted intestinal cell growth and migration, and decreased cell apoptosis, but inhibition of p70S6K by rapamycin negated these protective effects. In a mouse model of gut I/R, inhibition of p70S6K by rapamycin increased intestinal inflammation, permeability, and mucosal damage. Additionally, rapamycin treated animals had a significant increase in mortality. Our novel findings not only provide a new understanding of the molecular mechanisms contributing to intestinal I/R injury but have the potential to provide the basis for new therapeutic approaches to abrogate intestinal damage using mTOR or p70S6K analogues/inducers.

## Materials and Methods

### In vitro Model of Hypoxia/Reoxygenation

IEC-6, a non-transformed rat small intestinal cell line(CRL-1592) (ATCC, Rockvillle, MD, USA) was maintained in Dulbecco’s modified Eagle’s medium supplemented with 10% fetal bovine serum (FBS), containing 2 mM glutamine, 1% penicillin and 1% streptomycin at 37°C in an incubator under a humidified atmosphere of 5%CO_2_/95% air. Cells between passages 17 and 25 were used.

To model intestinal I/R in vitro, we performed in vitro experiments after hypoxia/reoxygenation. Cells were placed in a hypoxic chamber with 0.5% O_2_/5% CO_2_/94.5% N_2_ [55] followed by reoxygenation for indicated time points.

### Stimulation of p70S6K by Hypoxia/Reoxygenation in vitro

IEC-6 cells were serum-starved overnight under hypoxic conditions. FBS (10%) was added for the indicated time-points under normoxic conditions. Cells were harvested and Western blot analysis was performed for expression of phosphorylated p70S6K (p-p70S6K) protein.

### Cell Transfection

pRK7 (empty plasmid) and pRK7-HA-S6K1-WT (p70S6K expressing plasmid) (Addgene Inc. Cambridge, MA, USA) [56] were transient transfected into IEC-6 cells. All transfections were carried out with Lipofectamine 2000 (Invitrogen, Carlsbad, CA, USA). One day prior to transfection, cells in 1 ml of medium without antibiotics were plated to 12-well plate at density of 1×10^5^ cells per well. When cells were approximately 95% confluent, for each transfection, 1.6 µg of plasmid in 100 µl of Opti-MEM I reduced serum medium and 4 µl of Lipofectamine 2000 in 100 µl of Opti-MEM I reduced serum medium were incubated at room temperature for 5 min, respectively, and then mixed. After 20 min incubation at room temperature, the mixture was added to the well. The old medium was discarded and fresh medium without antibiotics was added after 6 h transfection.

### DNA Fragmentation in vitro

After transfection with plasmid for 24 h, cells in FBS-free medium in 12-well plates with vehicle (dimethyl sulfoxide) or rapamycin(100 nM) (a specific inhibitor of mTOR ) [57] were incubated under hypoxic conditions for 24 h and then reoxygenation for 6 h. The DNA fragmentation in cells was measured by a Cell Death Detection ELISA kit (Roche Diagnostics Corp. Indianapolis, IN, USA). Briefly, after treatment, the medium was discarded, cells were washed with phosphate buffered saline (PBS) then lysed. One hundred micromilliliter of cell lysate per well was transferred to the ELISA plate and measured in a microplate reader at 405/490 nm. The value was normalized by cell numbers in parallel experiments. Cells treated with empty pRK7 and incubated in normoxic conditions served as untreated controls.

### In vitro Cell Growth Assay

After transfection with plasmid for 24 h, cells (5×10^3^) were cultured overnight in 96-well plates with FBS-free medium under hypoxic conditions. Cells were then treated with 100 nM of rapamycin or vehicle for 30 min prior to adding10% FBS. Cells were cultured for 3 days in normoxic conditions. Each well was then added 20 µl of MTT (3-(4,5-dimethylthiazol-2-yl)-2,5-diphenyl tetrazolium bromide) (Sigma, Milwaukee, WI, USA) solution (5 mg/ml) and incubated at 37°C for 3 h. After incubation, each well was added with 200 µl dimethyl sulfoxide and measured at 570 nm using a microplate reader.

### Cell Migration in an in vitro Wounding Model

Plasmid transfected cells were cultured in 12-well plates with FBS-free medium overnight under hypoxic conditions, and then confluent cell monolayers were scraped using a 1-ml pipette tip and cells washed twice with PBS. Vehicle or rapamycin (100 nM) was added to each well. After 30 min, 10% of FBS added to each well. Wound areas before and after treatment were photographed using an inverted phase-contrast microscope with an attached camera and then analyzed using OPTIMAS 6.1 image analysis software (Optimas Corp., USA). The recovered surface area after 24 h was calculated [58].

### Mouse Model of Intestinal I/R

Animal procedures/protocols were approved by the University of Texas Houston Medical School Animal Welfare Committee (permit number: HSC-AWC-11-123). Male C57BL/6 mice (8–10 weeks old or 20–25 g) (Harlan Laboratories, Houston, TX, USA) were fasted overnight preceding experiments. After midline laparotomy under isoflurane-inhaled anesthetic, the celiac and superior mesenteric arteries were clamped at their origins for 1 h and reperfusion for 6 h. This time period was chosen as we have shown it produces reproducible gut injury without resultant mortality [59]. The animals were sacrificed and the intestine harvested for histologic examination, molecular, biological, biochemical and permeability studies. To understand the time-course of stimulation of p70S6K after reperfusion, another set of experiment was performed after 1 h of intestinal ischemia followed by 0 and 12 h of reperfusion. The 5-min time-point was chosen based on in vitro experiments showing p70S6K was stimulated by FBS within 5 min. For rapamycin treated animals, rapamycin (3 mg/kg) [60] or vehicle was given intraperitoneal 30 min prior to ischemia and animals were sacrificed 6 h after reperfusion. In the sham group, mice were underwent an identical procedure without superior mesenteric artery occlusion. For survival experiments, rapamycin (3 mg/kg) or vehicle was given intraperitoneal 30 min preceding ischemia. In these animals, the superior mesenteric artery was occluded for 75 min then the animals were observed for 7 days.

### Histopathologic Examination of the Intestinal Tissue

Intestinal tissues were fixed in formalin and embedded in paraffin. Tissue sections were deparaffinized and rehydraded, and then stained with hematoxylin and eosin. Histopathologic changes were evaluated with a light microscope in a blinded manner. The morphological criteria used for scoring on a 0 to 5 scale were according to Park/Chiu, where 5 is the most severely injured [61]–[63].

### TUNEL Immunofluorescence Staining for Apoptosis in the Intestinal Tissue

Formalin-fixed, paraffin-embedded intestinal sections were stained for apoptotic cells by the terminal deoxynucleotidyl transferase-mediated deoxyuridine triphosphate nick-end labeling (TUNEL) method using an In Situ Cell Death Detection Kit according to the manufacturer’s instructions (Roche, Indianapolis, IN). The apoptotic index was determined by dividing the number of positively stained cells by the total number of cells in the villus column and multiplying by 100. A minimum of 400 cells were counted.

### Myeloperoxidase (MPO) Activity Assessment of the Intestinal Tissue

Intestinal MPO activity reflects the extent of neutrophil infiltration in the intestine, was evaluated using Myeloperoxidase (MPO) Colorimetric Activity Assay Kit (BioVision Research Products, Mountain View, CA, USA) normalized to milligrams of protein. Protein concentration was measured by Bio-Rad Protein Assay (Bio-Rad Laboratories, Inc. Hercules, CA, USA).

### Naphthol AS-D Chloroacetate Esterase Staining of the Intestinal Tissue

Formalin-fixed, paraffin-embedded intestinal sections were stained with a Naphthol AS-D Chloroacetate Esterase Cytochemical Staining kit from Sigma Diagnostics (Milwaukee, WI ), which identifies specific leukocyte esterases. The number of positively stained cells in the intestinal mucosa was counted in 50 high-power fields (×400) per section under a microscope.

### Pro-inflammatory Cytokine Detection of the Serum and Intestinal Tissue

Levels of cytokines TNF-α, IL-6 and IL-1β in serum and intestinal tissues were measured by a commercial kit (Mouse Cytokine ELISA Strip I for Profiling 8 Cytokines kit,Signosis, Sunnyvale, CA, USA) according to manufacturers’ instructions.

### Intestinal Permeability Evaluation

To evaluate the intestinal permeability, the ex-vivo everted intestinal sac method was used [64]. Briefly, one end of intestinal sac was ligated in ice-cold modified Krebs-Henseleit bicarbonate buffer (KHBB, pH 7.4). The sac was then everted and filled with 1.5 ml of KHBB. The everted sac was then placed into 40 ml of KHBB containing fluorescein-isothiocyanate dextran probe (FD4, 4000 Da) (20 µg/ml) in a 50-ml beaker. The beaker was put into a water bath at 37°C and the buffer was continuously bubbled with a gas mixture of 95% oxygen and 5% carbon dioxide. Before placed the everted sac into water bath, a 1000 µl of sample was collected from the beaker as the initial mucosal side probe concentration. After incubation for 30 min, the length of the gut sac was measured. The fluid inside of the sac was collected to determine probe concentration of the serosal side. After centrifuged for 10 min at 1000 g at 4°C, a 100 µl supernatant from mucosal and serosal sides was diluted with 900 µl of PBS. The fluorescent signal was evaluated using a fluorescence plate reader with excitation/emission at 492/515 nm. Gut permeability was expressed as the mucosal-to-serosal clearance of probe calculated as previously reported [65].

### Immunohistochemical Staining of the Intestinal Tissue

The Dako-Cytomation EnVision + System-HRP (AEC) kit from Dako (Carpinteria, CA, USA) was employed for immunohistochemical staining, according to the manufacturer’s instructions. After deparaffinization and rehydration, the intestinal sections were covered using peroxidase block reagent and incubated at room temperature for 5 min. After wash with PBS, the sections were covered with phosphorylated p70S6 kinase (phospho-Ser411) antibody (GenWay Biotech, San Diego, CA, USA, 1∶100 dilution) and incubated 30 min at room temperature. Washing with PBS, the sections were incubated with peroxidase labeled polymer at room temperature for 30 min. The sections were added with the ready-to-use AEC + substrate-chromogen solution and incubated for 3–5 min after washing with PBS. The sections were then lightly counterstained in hematoxylin and mounted. In negative controls, PBS was used to replace primary antibody.

### Western Blot Analysis

IEC-6 cells and intestinal tissues were lysed using radioimmunoprecipitation assay buffer with additional protease inhibitors. Extracted proteins were quantified by the Bio-Rad Protein Assay. Electrophoresis of the proteins was carried out in a SDS-polyacrylamide gel. Proteins were blotted on Hybond-P membranes. After blocking using fat-free milk (5%) in tris buffered saline containing Tween 20 (0.1%) at room temperature for 1 h, the membranes were incubated with antibodies against p70S6K (p70 S6 kinase antibody and phospho-p70 S6 kinase (Thr421/Ser424) antibody) and β-actin (all purchased from Cell signaling, Danvers, MA, USA) overnight at 4°C. The blots were then incubated with ECL anti-rabbit IgG, horseradish peroxidase linked whole antibody (from donkey) for 1 h at room temperature. The blots were developed using ECL plus Western blotting detection system (GE Healthcare Biosciences, Piscataway, NJ, USA).

### Statistical Analysis

Data are expressed as mean ± SEM and were analyzed by one way analysis of variance. The statistical analysis comparing 2 groups was performed with nonparametric Mann-Whitney test. Tukey’s multiple group comparison test was used to compare individual group means. The Mantel–Haenzel log rank test was employed to performed survival comparisons. P values less than 0.05 were considered statistically significant.
